# Quantifying the risk of Zika virus spread in Asia during the 2015-16 epidemic in Latin America and the Caribbean: A modeling study

**DOI:** 10.1016/j.tmaid.2020.101562

**Published:** 2020

**Authors:** Xue Shi Luo, Natsuko Imai, Ilaria Dorigatti

**Affiliations:** a Imperial College London, St Mary's Campus, Norfolk Place, London, W2 1PG, UK; b MRC Centre for Global Infectious Disease Analysis, Department of Infectious Disease Epidemiology, Imperial College London, St Mary's Campus, Norfolk Place, London, W2 1PG, UK

**Keywords:** Zika virus, Asia, Southeastern, Latin America, Epidemics, Disease transmission, Infectious, CDC, Centers for Disease Control and Prevention, CI, Confidence interval, DENV, Dengue virus, LAC, Latin America and the Caribbean, OAG, Official Aviation Guide, PAHO, Pan American Health Organization, US, United States, SEA, Southeast Asia, ZIKV, Zika virus

## Abstract

**Background:**

No large-scale Zika epidemic has been observed to date in Southeast Asia following the 2015-16 Latin American and the Caribbean epidemic. One hypothesis is Southeast Asian populations’ partial immunity to Zika.

**Method:**

We estimated the two conditions for a Zika outbreak emergence in Southeast Asia: (i) the risk of Zika introduction from Latin America and the Caribbean and, (ii) the risk of autochthonous transmission under varying assumptions on population immunity. We also validated the model used to estimate the risk of introduction by comparing the estimated number of Zika seeds introduced into the United States with case counts reported by the Centers for Disease Control and Prevention (CDC).

**Results:**

There was good agreement between our estimates and case counts reported by the CDC. We thus applied the model to Southeast Asia and estimated that, on average, 1–10 seeds were introduced into Indonesia, Malaysia, the Philippines, Singapore, Thailand and Vietnam. We also found increasing population immunity levels from 0 to 90% reduced probability of autochthonous transmission by 40% and increasing individual variation in transmission further reduced the outbreak probability.

**Conclusions:**

Population immunity, combined with heterogeneity in transmission, can explain why no large-scale outbreak was observed in Southeast Asia during the 2015-16 epidemic.

## 1 Introduction

Zika virus (ZIKV) is a *Flavivirus* first isolated in 1947 from a rhesus monkey in the Zika Forest, Uganda [1]. In May 2015, autochthonous ZIKV transmission was reported in Northeast Brazil [2]. As ZIKV rapidly spread across Latin America and the Caribbean (LAC), its high disease burden became apparent. In February 2016, the World Health Organization declared the growing epidemic as a Public Health Emergency of International Concern [3].

While ZIKV can be spread by infected blood [4], vertical [5] and sexual transmission [6], humans are primarily infected through the bites of infected *Aedes* mosquitoes [7]. Given the ease and speed of international air-travel, a global Zika epidemic in 2015–16 was feared as viremic travelers could bring the virus from LAC to distant locations with local competent mosquito vectors.

Particularly, a large-scale epidemic was feared to take off in Southeast Asia (SEA) following the 2015-16 LAC epidemic [8,9]. In addition to having climatic conditions conducive for autochthonous transmission, SEA has a very high prevalence of competent *Aedes* vectors [10]. However, despite these favorable conditions, a large-scale epidemic has not been observed in the region to date [9].

Although Singapore had recorded the importation of one Zika case from Brazil in May 2016 and experienced an outbreak with 455 confirmed autochthonous cases from August to November 2016, subsequent phylogenetic studies revealed that the ZIKV that had caused Singapore's outbreak was more closely related to the virus circulating in SEA before 2007 rather than the virus responsible for the LAC epidemic [10]. Questions remain as to why a large-scale epidemic has not been observed in SEA despite the high prevalence of competent *Aedes* vectors and a conducive climate for ZIKV spread [11].

One hypothesis is that some outbreaks might have occurred but went undetected or underreported [12]. Detection of ZIKV can be difficult due to asymptomatic infection or the similarity of ZIKV symptoms to those of other flaviviruses, especially dengue virus (DENV) which is endemic in SEA. Undetected ZIKV and/or misdiagnosis may also have been exacerbated by the limitations of the surveillance systems in some Southeast Asian countries [13]. In addition, some countries may not have disclosed ZIKV-related information for fear of negative impacts on tourism [14].

Other than suboptimal detection and/or reporting, another hypothesis is that Southeast Asian populations may have partial immunity to ZIKV. ZIKV was first isolated in Malaysia in the 1960s and there have been reports of sporadic ZIKV infections in SEA since the 1950s [15]. Previous serological studies have also confirmed past circulation of ZIKV in SEA [[16], [17], [18]], implying that some proportion of the populations may therefore be immune. Additional evidence supporting the immunological hypothesis includes the role of cross-protective immunity [11]. Recent studies [19,20] have demonstrated that pre-exposure to SEA-endemic flaviviruses such as DENV is associated with a lower risk of ZIKV infection, thus suggesting that endemic or hyperendemic DENV transmission in SEA can provide high levels of cross-protection against ZIKV infection, thereby decreasing the risk of local ZIKV transmission.

Despite the hypothesized resistance of Southeast Asian populations to ZIKV infection, frequent introductions due to globalization and international travel represent a serious risk for the establishment of endemic ZIKV circulation in SEA, which would have detrimental health and economic consequences for the whole region. As the extent of ZIKV introduction from LAC and autochthonous ZIKV transmission in SEA remains unknown, we implemented the method developed by Dorigatti et al. [21] to estimate the number of ZIKV infections expected to be introduced into SEA during the 2015-16 LAC epidemic. We validated this method by comparing the estimated number of ZIKV cases introduced into the United States (US) during the 2015-16 LAC epidemic with case counts reported by the US Centers for Disease Control and Prevention (CDC) [22]. We then quantified the risk of ZIKV introduction into SEA and used a mathematical model to estimate the probability of autochthonous transmission following introduction, assuming varying levels of ZIKV immunity levels in Southeast Asian populations. These estimates provide quantitative information on the risk of disease introduction and local spread, and in real-time outbreak context can inform response planning. For instance, estimates can help policy decision makers evaluate the need for and the optimal deployment of interventions (e.g. travel restriction, symptom screening or proof of vaccination at specific ports of entry), which is particularly important in resource-constrained settings.

## 2 Methods

There are two necessary conditions for international ZIKV spread to occur, (i) the introduction of ZIKV into unaffected countries, and (ii) autochthonous ZIKV transmission after introduction. ZIKV can be introduced into an unaffected country via ‘importation’ and/or ‘exportation’. The term ‘importation’ refers to tourists who were infected during their travels in the affected areas and brought the virus to the unaffected areas during their incubation or infectious period. The term ‘exportation’ refers to infected residents from affected areas traveling to unaffected areas during their incubation or infectious period. The sum of the number of importations and exportations i.e. the number of introduced seeds thus represent the risk of ZIKV introduction into an unaffected country. The magnitude of this risk depends on the degree of interconnectivity between the affected and unaffected countries, with larger-scale connectivity producing a higher risk [23]. In contrast, the risk of autochthonous transmission is determined by local factors such as population susceptibility, presence of competent vectors and climatic suitability [24].

### 2.1 Estimating risk of ZIKV introduction

We adopted the method developed by Dorigatti et al. [21] and applied it to the Official Aviation Guide (OAG) travel volume data [25]. We used estimates of the point of ticket sale as proxy for travelers’ residency to estimate the risk of ZIKV introduction into the US and SEA during the 2015-16 LAC epidemic (see Supplementary Material).

#### 2.1.1 Model validation

We evaluated the model's predictive performance by estimating the number of introductions from the 38 Zika-affected LAC countries into each US state. We assumed that the main form of travel between LAC and the US was by air and hence only used air-travel data as inputs. We also assumed that symptomatic ZIKV cases had the same probability of travel as healthy individuals. The number of travelers between each LAC country and each US state, including information on the country of ticket sale, was obtained from the OAG database [25], which comprised airport-level origin-destination estimates on the volume of travelers between airports. We then aggregated the number of travelers for months that fell within the respective LAC country's epidemic time window (i.e. the number of days between the time of symptom onset of the first and last confirmed cases). The population size, number of ZIKV cases and epidemic time window for each LAC country were extracted from the Pan American Health Organization (PAHO) weekly database [26] (Table 1). A summary of other parameters and their data sources, including the average length of stay by US travelers in all LAC countries [27,28], and intrinsic incubation period and human infectious period [[29], [30], [31]] is given in Table 2.

**Table 1:** Parameterization used in the model developed by Dorigatti et al. [21] to estimate the risk of Zika virus introduction.

Latin America and Caribbean countries	Population size	Date of first known case	Date of last known case	Number of cases
Argentina	40,117,000	2016-01-10	2016-05-28	1,632
Aruba	110,000	2016-01-03	2016-11-12	693
Antigua & Barbuda	91,295	2016-08-07	2016-11-19	469
Barbados	283,000	2015-12-20	2016-11-12	745
Belize	369,000	2016-01-10	2016-11-26	781
Bolivia	10,520,000	2015-12-27	2016-11-19	881
Brazil	48,218,000	2015-01-04	2016-11-12	314,468
Caiman Islands	56,732	2016-01-03	2016-12-03	226
Colombia	48,585,685	2015-09-20	2016-12-24	103,175
Costa Rica	4,937,455	2016-02-14	2016-11-26	1,553
Dominica	71,000	2016-01-03	2016-05-07	231
Dominican Republic	9,980,000	2016-01-03	2016-09-17	5,157
Ecuador	16,279,000	2015-12-27	2016-10-01	3,516
El Salvador	6,460,000	2015-10-18	2016-11-26	11,461
French Guyana	262,000	2016-01-03	2016-10-15	10,344
Guadeloupe	405,000	2016-01-17	2016-10-15	30,777
Guatemala	16,176,000	2015-11-15	2016-10-08	3,319
Guiana	747,000	2015-12-27	2016-07-30	39
Haiti	10,994,000	2015-10-18	2016-08-13	2,986
Honduras	8,950,000	2015-12-20	2016-09-24	31,876
Jamaica	2,729,000	2015-11-22	2016-10-29	6,536
Martinique	383,000	2015-12-27	2016-10-15	36,622
Mexico	121,006,000	2015-10-18	2016-11-12	6,756
Nicaragua	6,514,000	2016-01-24	2016-05-21	207
Panama	3,764,000	2015-11-22	2016-11-26	2,948
Paraguay	7,003,000	2015-10-25	2016-11-26	646
Peru	30,380,000	2016-05-08	2016-12-17	1,663
Puerto Rico	3,508,000	2016-01-03	2016-12-10	35,706
St. Barthélemy	9,625	2015-12-27	2016-12-03	952
St. Kitts & Nevis	46,398	2016-06-26	2016-11-05	567
St. Martin	35,684	2016-01-17	2016-11-26	3,016
St. Vincent & Grenadines	110,167	2016-01-24	2016-10-29	585
Sint Maarten	39,000	2015-12-27	2016-10-01	230
Suriname	560,000	2015-09-20	2016-11-26	3,529
Trinidad &Tobago	1,357,000	2016-02-07	2016-10-08	658
Virgin Islands UK	105,000	2016-06-26	2016-09-17	104
Virgin Islands US	105,000	2016-01-10	2016-11-19	1,666
Venezuela	30,620,000	2016-01-03	2016-11-26	58,657

**Table 2:** Parameterization used in the model developed by Dorigatti et al. [21] to estimate the risk of disease introduction, their definitions and data sources.

Parameter	Definition	Data source (references)
*T*_*D→*__*O*_	Number of resident travelers in unaffected areas traveling to Latin America and the Caribbean during the epidemic time window (residency was defined by the point of ticket sale)	OAG dataset [25]
*T*_*O→*__*D*_	Number of resident travelers in Latin America and the Caribbean traveling to unaffected areas during the epidemic time window (residency was defined by the point of ticket sale)	
*λ*	Per capita risk of infection of international travelers during their stay in Latin America and the Caribbean	Refer to Equation 1.2
*p*_*H*_	Probability of travelers returning home while incubating or infectious	Refer to Equation 1.3
*p*_*O→*__*D*_	Per capita probability that a resident of Latin America and the Caribbean travels to unaffected areas	Refer to Equation 1.5
*p*_*I*_	Probability that an infected case incubates or is infectious in the epidemic time window	Refer to Equation 1.6
*N*	Number of cases in Latin America and the Caribbean	PAHO weekly database [26]
*pop*_*O*_	Population size of Latin America and the Caribbean	
*W*	Epidemic time window (days)	
*L*	Average length of stay of travelers visiting Latin America and the Caribbean (days)	U.S. Department of Commerce [ [27,28]]
*T*_*E*_	Intrinsic incubation period (days)	Publication [29]
*T*_*I*_	Human infectious period (days)	Publication [ [30,31]]

Uncertainty around the expected number of introduced seeds was obtained by sampling 10,000 realizations from the incubation and infectious period distributions to compute the 95% confidence interval (CI). We then compared the estimated number of introductions into each US state with the case counts reported by the CDC in 2015–16 [22]. The CDC-reported case counts for each state included travelers returning from affected areas (94.8%), cases acquired through local transmission in the US (4.3%), and cases acquired through other routes e.g. sexual transmission (0.9%). In this analysis we discarded the cases acquired through local transmission but included the number of cases who acquired ZIKV through other routes as information on the states of residence for these cases were not reported.

We calculated Spearman's rank correlation and Pearson's correlation coefficient between the estimated mean number of introductions and the CDC data [22]. We also evaluated the strength of the association using linear regression.

#### 2.1.2 Application of model to SEA

After validating the model using the case counts reported by the CDC, we applied it to SEA to estimate the number of potential seeds introduced from LAC into each Southeast Asian country during the 2015-16 LAC epidemic. Table 2 summarizes the data sources of the inputs (e.g. the number of travelers between LAC and SEA, the intrinsic incubation period, the human infectious period, the population size and epidemic time window for each LAC country) used in the model. When applying the model to SEA, we assumed that asymptomatic infections represent 62% of all ZIKV infections [32] and scaled the case counts reported by PAHO (which represent symptomatic infections) by 2.63 (i.e. 1/(1–0.62)) to estimate the total number of ZIKV infections comprising both symptomatic and asymptomatic infections. We also assumed that the average number of days Asian travelers spent overseas (7 days) [33] represented the average length of stay of travelers from SEA in LAC countries.

### 2.2 Estimating risk of autochthonous ZIKV transmission

We adopted the method described in Johansson et al. [34] to quantify the risk of autochthonous transmission after introduction (see Supplementary Material), and explored three different transmissibility scenarios (low, moderate and high) in the parameterization of: (i) the average number of infectious vectors produced per infectious human (R_0_^HV^); (ii) the average number of infectious humans produced per infectious vector (R_0_^VH^); and (iii) the basic reproduction number (R_0_).

A summary of the parameters used to estimate the risk of autochthonous ZIKV transmission and their data sources, such as the number of bites per mosquito [35], can be found in Table 3. As *Aedes aegypti* is the main vector of ZIKV in SEA [36], we parameterized the model using empirical SEA-specific and *Aedes aegypti*-related values whenever available. For example, assuming 50% of pupae are female, we assumed that the number of female mosquitoes per person in SEA was half the reported number of *Aedes aegypti* pupae per person in Thailand [37]. We followed Mordecai et al. [35] and estimated the average vector longevity to be 27 days at an average SEA temperature of about 27 °C [38]. We derived the average proportion of vectors surviving the extrinsic incubation period by assuming an exponential distribution (i.e. $e^{\left(-\frac{3}{27}\right)}$, given the mortality rate of 1/27 days^−1^ [35]) and an extrinsic incubation period of 3 days [36].

**Table 3:** Parameterization used to model the three transmissibility scenarios (low, moderate and high) to estimate the risk of autochthonous Zika virus transmission and their data sources.

Transmissibility scenario				
Parameter	Low	Moderate	High	Data source
Number of female mosquitoes per person	0.85	0.85	0.85	[37]
Number of bites per mosquito per day	0.3	0.3	0.3	[35]
Average duration of human infectious period (days)	5	5	5	[30,31]
Average vector longevity (days)	27	27	27	[35]
Average proportion of vectors surviving the extrinsic incubation period	0.89	0.89	0.89	[35,36]
Effective transmission rate from human to vector	0.8	0.9	1	[39]
Effective transmission rate from vector to human	0.2	0.4	0.6	–

Following a recent study by Uraki et al. [39], where 80–100% of the *Aedes aegypti* mosquitoes fed on ZIKV-infected mice was infected, we assumed that the effective transmission rate from humans to vectors was 0.8, 0.9 and 1 for the low, moderate, and high transmissibility scenario respectively. As the effective transmission rate from vectors to humans is not measurable due to ethical reasons, we arbitrarily set it as 0.2, 0.4 and 0.6 respectively for the low, moderate and high transmissibility scenarios to produce R_0_ values that are in line with the estimates observed in LAC [40] and the Pacific Islands [41] (Table 4).

**Table 4:** Reproduction numbers computed for each transmissibility scenario (low, moderate, high) if populations were fully susceptible to Zika virus.

Transmissibility scenario			
Parameter	Low	Moderate	High
Average number of infectious vectors produced per infectious human, R_0_^HV^	0.91	1.02	1.13
Average number of infectious humans produced per infectious vector, R_0_^VH^	1.62	3.24	4.86
Basic reproduction number, R_0_	1.47	3.31	5.51

We then used the branching process approach described in Johansson et al. [34] to estimate the probability of autochthonous transmission, having assumed a negative binomial probability-generating function with dispersion parameter fixed at *k* = 0.1 [[41], [42], [43], [44]]. Sensitivity analysis on the impact of the assumed level of dispersion (parameter *k*) on the estimates of the probability of autochthonous transmission was performed.

For Southeast Asian countries estimated to have received at least one potential seed from LAC, we quantified the probability of autochthonous transmission assuming the independent introduction of the estimated mean and relative 95% CI number of seeds. All estimates were calculated assuming varying ZIKV immunity levels in the Southeast Asian populations from 0 to 100%.

All analyses were performed using the R package *epiflows* [45] and using R version 3.5.0 [46].

## 3 Results

**Fig. 1:**
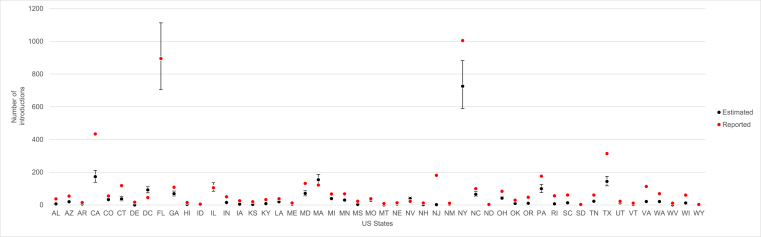
Estimated mean number of ZIKV introductions from Latin America and Caribbean into each state in the United States (black dots) and ZIKV case counts from the United States Centers for Disease Control and Prevention dataset (red dots). Black bars denote the 95% confidence interval. AL: Alabama, AZ: Arizona, AR: Arkansas, CA: California, CO: Colorado, CT: Connecticut, DE: Delaware, DC: District of Columbia, FL: Florida, GA: Georgia, HI: Hawaii, ID: Idaho, IL: Illinois, IN: Indiana, IA: Iowa, KS: Kansas, KY: Kentucky, LA: Louisiana, ME: Maine, MD: Maryland, MA: Massachusetts, MI: Michigan, MN: Minnesota, MS: Mississippi, MO: Missouri, MT: Montana, NE: Nebraska, NV: Nevada, NH: New Hampshire, NJ: New Jersey, NM: New Mexico, NY: New York, NC: North Carolina, ND: North Dakota, OH: Ohio, OK: Oklahoma, OR: Oregon, PA: Pennsylvania, RI: Rhode Island, SC: South Carolina, SD: South Dakota, TN: Tennessee, TX: Texas, UT: Utah, VT: Vermont, VA: Virginia, WA: Washington, WV: West Virginia, WI: Wisconsin, WY: Wyoming. [color should be used in print]. (For interpretation of the references to color in this figure legend, the reader is referred to the Web version of this article.)

Fig. 1 and Supplementary Table 2 show a comparison between the reported number of introductions into the US and the expected number of introductions obtained with the method developed by Dorigatti et al. [21]. Both the Pearson's correlation coefficient (0.95, 95% CI 0.92–0.97) and the Spearman's rank correlation (0.83, 95% CI 0.72–0.90) revealed a strong positive correlation between the estimated mean number of introductions and the reported case counts. We found an estimated slope of 0.80 (95% CI 0.72–0.87) between the reported ZIKV case counts and the estimated number of ZIKV introductions (Supplementary Fig. 1), showing good agreement and an average underestimation of 20% (95% CI 13–28%).

**Fig. 2:**
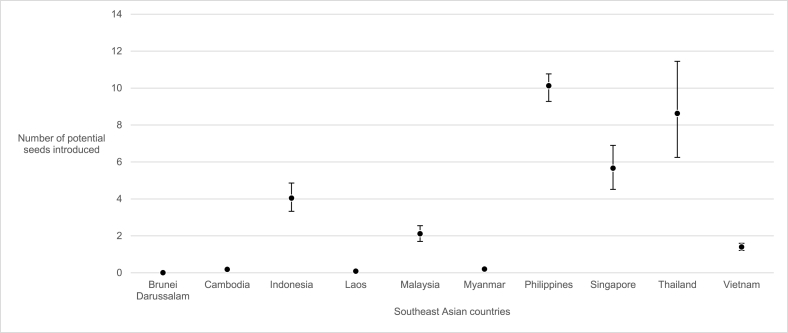
Estimated mean number of potential seeds introduced from Latin America and Caribbean into each Southeast Asian country (black dots). Black bars denote the 95% confidence interval.

We estimated that during the 2015-16 LAC epidemic, the Philippines received the highest number (10 seeds, 95% CI 9–11) of potential seeds (importations and exportations), followed by Thailand (9 seeds, 95% CI 6–11) and Singapore (6 seeds, 95% CI 5–7). In contrast, we estimated that there was no seed introduction into Brunei Darussalam, Cambodia, Laos or Myanmar (Fig. 2).

**Fig. 3:**
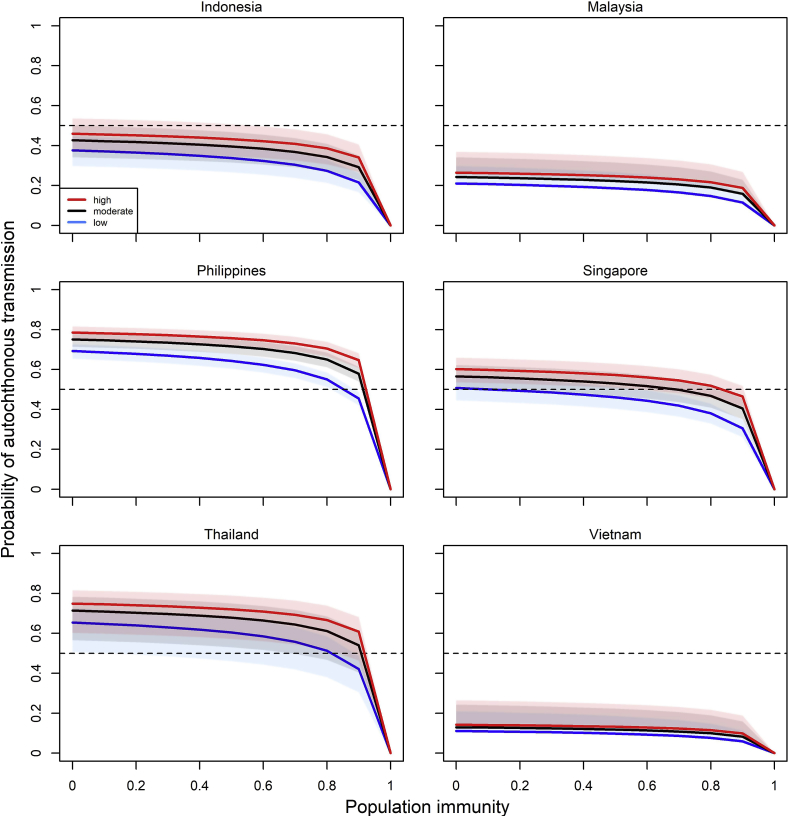
Estimated probabilities of autochthonous transmission in Indonesia, Malaysia, the Philippines, Singapore, Thailand and Vietnam assuming independent introductions of the estimated mean number of potential seeds at varying population immunity levels. Lines denote the estimated average probabilities of autochthonous transmission and the shaded areas denote the 95% confidence interval. Red, black and blue colors respectively denote the high, moderate and low transmissibility scenarios. *[color should be used in print].* (For interpretation of the references to color in this figure legend, the reader is referred to the Web version of this article.)

We then used the estimated number of ZIKV introductions from LAC into SEA to estimate the probability of autochthonous transmission for Indonesia, Malaysia, the Philippines, Singapore, Thailand and Vietnam (the countries estimated to have received at least one potential seed from LAC during the 2015-16 LAC epidemic), under the three (low, moderate and high) transmissibility scenarios. We found that in these six countries, increasing population immunity levels from 0 to 90% reduced the probabilities of autochthonous transmission by an average of 41%, 40%, and 37% in the low, moderate and high transmissibility scenarios respectively (Fig. 3 and Table 5). Out of the six countries, Indonesia, Malaysia and Vietnam had a lower-than-chance (<50%) probability of autochthonous transmission even when their populations were assumed to be fully susceptible. For the Philippines and Thailand, the probabilities of autochthonous transmission only dropped below 50% when the population immunity level was approximately 80% or higher across all transmissibility scenarios. For Singapore, the estimated probability of autochthonous transmission dropped below 50% when the population immunity was more than 20% in the low, 70% in the moderate and 80% in the high transmissibility scenario (Fig. 3).

**Table 5:** Percentage change in probability of autochthonous transmission if the estimated mean number of seeds were independently introduced in each Southeast Asian country and the population immunity was increased from 0 to 90%.

Southeast Asian country	Probability of autochthonous transmission if population is fully susceptible	Probability of autochthonous transmission if 90% of population is immune to Zika virus		Percentage difference in probability when population immunity was increased from 0 to 90%
Low transmissibility scenario				
Indonesia	0.38	0.22	−42%	
Malaysia	0.21	0.11	−48%	
The Philippines	0.69	0.46	−33%	
Singapore	0.51	0.31	−39%	
Thailand	0.65	0.42	−35%	
Vietnam	0.11	0.06	−45%	
Average			−41%	
Moderate transmissibility scenario				
Indonesia	0.40	0.23	−43%	
Malaysia	0.22	0.12	−45%	
The Philippines	0.72	0.48	−33%	
Singapore	0.53	0.33	−38%	
Thailand	0.68	0.45	−34%	
Vietnam	0.12	0.06	−50%	
Average			−40%	
High transmissibility scenario				
Indonesia	0.41	0.25	−39%	
Malaysia	0.23	0.13	−43%	
The Philippines	0.74	0.51	−31%	
Singapore	0.55	0.35	−36%	
Thailand	0.70	0.47	−33%	
Vietnam	0.12	0.07	−42%	
Average			−37%	

In the sensitivity analysis, we found that increasing heterogeneity in the offspring distribution (i.e. when the dispersion parameter *k* is smaller) is associated with a decreasing risk of autochthonous transmission (Supplementary Figs. 2–4).

## 4 Discussion

We validated the method developed by Dorigatti et al. [21] against the ZIKV case count data reported by the CDC and applied it to estimate the number of ZIKV introductions into SEA, where to date, a large ZIKV outbreak has not yet been observed. We found that the method developed by Dorigatti et al. [21] can generally estimate the risk of disease introduction accurately (Fig. 1 and Supplementary Fig. 1). As it was not possible to exclude the number of cases acquired through non-vector borne transmission routes (e.g. sexual transmission) from the total case counts reported by the CDC, the model is expected to slightly underestimate the data reported by the CDC. In addition, the slight discrepancy observed between the model predictions and the CDC estimates in California, New York and Texas (Fig. 1), which are the top three US states with the largest share and volume of US travelers [27,28], could be due to limitations such as the use of aggregated origin-destination flight data that by construction do not capture multi-legged travel itineraries. Another possible explanation is that the model does not account for other forms of travel (e.g. by land) that certainly contributed to disease introduction. Underestimating the risk of disease importation may underestimate the risk of autochthonous transmission and the efforts needed to prevent disease introduction and the control of local onward transmission. On the other hand, overestimation of the number of introductions may suggest the need of interventions that are above the actual level of risk. While overall, the estimates obtained in this analysis tend to slightly underestimate the risk of disease introduction in the United States, the estimated relationship between the observed and estimated number of introductions can be used to correct the predictions. The availability of data on travel-related case importations is of paramount importance to test, validate and improve models and estimates.

When we applied the model to SEA, we found that in general, the estimated numbers of potential seeds introduced from LAC into SEA were substantially lower than the estimated number of introductions into the US. This is in concordance with a previous study [8] that concluded that the risk of ZIKV introduction was highest for the US during the 2015-16 LAC epidemic and less so for SEA due to smaller air traffic flows between SEA and LAC [8].

We found low support for ZIKV introduction from LAC into Brunei Darussalam, Cambodia, Laos and Myanmar, which likely explains why no outbreak has been observed in these countries. As of 2018, there has been no reported Zika case in Brunei Darussalam [47]. In Cambodia, Laos and Myanmar, there was only one reported ZIKV infection in 2016 [[48], [49], [50]] but it is unclear if the case resulted from travelers introduced from LAC or from elsewhere.

We estimated that Indonesia, Malaysia and Vietnam received an average of 4 (95% CI 3–5), 2 (95% CI 2–3) and 1 (95% CI 1–2) potential seeds from LAC respectively, producing probabilities of autochthonous transmission below 50% across all transmissibility scenarios using the baseline parametrization of overdispersion (*k* = 0.1). In contrast, we estimated that the Philippines, Thailand and Singapore respectively received an average of 10 (95% CI 9–11), 9 (95% CI 6–11) and 6 (95% CI 5–7) potential seeds from LAC. Under the baseline parameterization of the overdispersion parameter (*k* = 0.1), the estimated number of introduced seeds into the Philippines and Thailand produced probabilities of autochthonous transmission which decrease from around 80%–50% as population immunity increases up to 80% across all transmissibility scenarios. For Singapore, the probability of autochthonous transmission decreases from 60% to 40% as population immunity increases up to 80%.

Despite our estimates of the probability of autochthonous transmission suggesting that the Philippines, Thailand and Singapore had potential for ZIKV spread, no large-scale epidemic has been observed. There are several plausible explanations for this. Firstly, this may be due to spatiotemporal heterogeneity as well as individual-level heterogeneity in transmission in these countries. Our sensitivity analysis showed that higher levels of overdispersion (*k* = 0.01 and *k* = 0.05) substantially reduced the risk of autochthonous transmission (Supplementary Figs. 2–4). Secondly, in this analysis we assumed no interventions, while many countries in SEA deployed vector control and community engagement measures in response to the 2015-16 LAC epidemic [51]. These efforts might have further reduced ZIKV transmissibility and mitigated the risk of ZIKV autochthonous transmission in SEA during the 2015-16 LAC epidemic. Thirdly, because most ZIKV infections are asymptomatic or mild, it is also possible that low levels of autochthonous ZIKV transmission occurred in SEA that went undetected or underreported. For example, it was reported that five confirmed ZIKV cases in Korea had travel history to SEA, which suggests the possibility of autochthonous ZIKV transmission in SEA [52].

This study has several limitations. When estimating the number of ZIKV seeds introduced into the US and SEA, we assumed a constant average length of stay in LAC countries and that travelers were exposed to the same risk of infection as residents of the affected areas. While homogeneity in the average length of stay and exposure to ZIKV is certainly a simplification, the estimated numbers of ZIKV introductions into the US were shown to be consistent with data reported by the CDC. Additional details (e.g. on travelers' final destinations, activities and behaviors as well as ZIKV's R_0_ estimates at fine spatiotemporal resolution) would have allowed refinement of the model parameterization and further improvement in the model's performance. We also approximated travelers' residency with the country of ticket sale provided in the OAG dataset. For instance, we assumed all airline tickets sold in a specific country were purchased by the country residents rather than international travelers. In future analyses, it would be interesting to explore how the point of sale information compares with nationality estimates where available. Thirdly, in the estimation of the probability of autochthonous transmission, we assumed homogeneity in the parameterization of the mosquito traits (e.g. vector density and biting rate) and hence in ZIKV's R_0_ across the Southeast Asian countries. Although there is evidence to suggest that ZIKV transmission varies temporally and spatially depending on climatic, ecological and socio-demographic factors (such as temperature, land-use and housing characteristics [53]), adding such details in the model would be beyond the reach of currently available data. Additionally, we assumed the same degree of overdispersion (*k* = 0.1) for the probability-generating functions for both humans and vectors and parameterized it according to estimates obtained in previous studies for vector-borne diseases [[41], [42], [43], [44]]. While it is possible that overdispersion in the human and vector distributions differs, sensitivity analysis on the level of overdispersion shows that, in line with previous observations [44], increasing heterogeneity in the offspring distribution is associated with a decreasing risk of autochthonous transmission (Supplementary Figs. 2–4).

Despite these limitations, this study serves as an important step towards an improved understanding of the risk of ZIKV spread from LAC to SEA. We first demonstrated that the model developed by Dorigatti et al. [21] applied to OAG travel volume data and using the point of sale as predictor of residency can be used to predict the risk of disease introduction. The model may therefore serve as an important new tool for outbreak surveillance that can inform response planning in future outbreaks. Rather than simply using travel volume as a proxy to determine the risk of disease introduction, it is of greater public health relevance to quantify the risk of disease introduction by estimating the actual number of travelers potentially capable of seeding autochthonous transmission. In addition to identifying locations where potential seeds are most likely to be introduced, quantitative estimates of the expected number of introduced seeds can inform prevention and mitigation efforts such as determining the implementation of travel restrictions, requiring proof of vaccination and the use of diagnostics at the ports of entry. If resources are constrained and prioritization is needed, these interventions could potentially be targeted at specific ports of entry (e.g. those receiving the largest travel volumes from the affected locations).

Given that the actual extent of ZIKV introduction from LAC and the risk of autochthonous ZIKV transmission in SEA were unknown, we retrospectively analyzed the risk of ZIKV spread and explored how the risk is affected by different assumptions on population immunity. Our findings suggest that not only population immunity (e.g. due to previous ZIKV infection or cross-protection conferred by previous dengue infections) but also heterogeneity (or individual variation) in transmission can explain why a large-scale ZIKV epidemic has not been observed in SEA during the 2015-16 LAC epidemic. The methods used in this study can be applied to other emerging or re-emerging diseases such as yellow fever, which is also absent in SEA. In addition, the model can be applied to other countries, such as India [8] or China [54,55], which were also predicted to be at high risk of ZIKV spread, or to entire regions such as Africa, which also had sporadic reports of ZIKV infections before the 2015-16 LAC epidemic [56].

Moving forward, it will be interesting to explore the impact of spatiotemporal heterogeneity in transmission on the outbreak probability. For instance, the mathematical model presented in this study can be combined with high-resolution vector abundance and climatic suitability estimates. Furthermore, mathematical models applied to ZIKV seroprevalence data could potentially be used to test the different assumptions behind the apparent lack of sustained ZIKV transmission in SEA. Comparisons between the risk of ZIKV spread in different regions may also provide further insights into the international spread of ZIKV during the 2015-16 LAC epidemic.

## Funding sources

This work is jointly funded by the UK Medical Research Council (MRC) and the UK Department for International Development (DFID) under the MRC/DFID Concordat agreement and is also part of the EDCTP2 programme supported by the European Union. I.D. also acknowledges research funding from an Imperial College Junior Research Fellowship and a Sir Henry Dale Fellowship funded by the Royal Society and Wellcome Trust [grant 213494/Z/18/Z].

## CDC disclaimer

The findings and conclusions in this report are those of the authors and do not necessarily represent the official position of the United States Centers for Disease Control and Prevention. Use of trade names and commercial sources is for identification only and does not imply endorsement by the United States Centers for Disease Control and Prevention.

## Data availability

The air passenger data used in this study were purchased from OAG Aviation Worldwide Ltd. These data are proprietary and used under the United States Centers for Disease Control and Prevention licenses for the current study and so are not publicly available. The authors are available to share the air passenger data upon reasonable request and with the permission of OAG Aviation Worldwide Ltd.

## CRediT authorship contribution statement

**Xue Shi Luo:** Software, Formal analysis, Writing - original draft. **Natsuko Imai:** Conceptualization, Methodology, Writing - review & editing, Supervision. **Ilaria Dorigatti:** Conceptualization, Methodology, Software, Resources, Data curation, Writing - review & editing, Visualization, Supervision.

## Declaration of competing interest

None of the authors have other association that might pose a conflict of interest.
