# Supplementary material for: Quantifying the risk of Zika virus spread in Asia during the 2015-16 epidemic in Latin America and the Caribbean: A modeling study
Source: Travel Med Infect Dis. 2020 Jan-Feb;33:101562. doi: 10.1016/j.tmaid.2020.101562 (PMC7049897; doi:10.1016/j.tmaid.2020.101562)
Supplement: Multimedia component 1 [file mmc1.docx]

**Supplementary Material**

1. **METHODS**
   1. **Estimating the risk of ZIKV introduction**

The mathematical model developed by Dorigatti et al. [21] integrates epidemiological data with travel data to quantify the risk of disease introduction. It models the two ways a disease can be introduced into an unaffected country, i.e. through importation and exportation, as described below.

#### Importation

Let *O* represent the affected areas (*O* for origin of disease) and *D* represent the unaffected areas (*D* for destination). The number of importations refers to the number of tourists infected during their stay in *O* and then returning to their home country *D* before they recover. It is derived by multiplying the number of travelers from *D* to *O* during the epidemic time window (*T_D🡪 O_*), the per capita risk of infection (*λ*) during their stay in *O*, and the probability of returning home while incubating or infectious (*p_H_*).

| $Number of importations= T_{D\to O}\lambda p_{H}$ | (1.1) |
| --- | --- |

The parameter *λ* is in turn estimated by multiplying the number of cases (*N*) in *O* with the average length of stay (*L*) by travelers visiting *O*, divided by the population size of *O* (*pop_O_*) and the epidemic time window (*W*).

| $\lambda=\frac{N L}{{pop}_{O} W}$ | (1.2) |
| --- | --- |

On the other hand, the probability of returning home while incubating or infectious, *p_H,_* depends on the intrinsic incubation period (*T_E_*), human infectious period (*T_I_*) and *L*.

| $p_{H}= minimum(\frac{T_{E +}T_{I}}{L} ,1)$ | (1.3) |
| --- | --- |

#### Exportation

The number of exportations refers to the number of infected residents of *O* traveling to *D* while incubating or infectious during the epidemic time window. This can be derived by multiplying the number of cases, *N,* with the per capita probability that a resident of *O* travels to *D* (*p_O🡪 D_*), and the probability that a case is incubating or infectious in the epidemic time window (*p_I_*).

| $Number of exportations=N p_{O\to D}p_{I}$ | (1.4) |
| --- | --- |

The parameter *p_O🡪 D_* is in turn obtained by dividing the number of travelers from *O* to *D* during the epidemic time window (*T_O🡪 D_*) by *pop_O_*.

| $p_{O\to D}=\frac{T_{O D}}{{pop}_{O}}$ | (1.5) |
| --- | --- |

Similar to the calculations for importation, *p_I_* depends on *T_E_*, *T_I_* and *W*.

| $p_{I}= minimum(\frac{T_{E +}T_{I}}{W} ,1)$ | (1.6) |
| --- | --- |

The output of the model developed by Dorigatti et al. [21] is the total number of travelers potentially capable of seeding autochthonous transmission introduced from *O* into *D*, which is the sum of the number of importations and exportations.

#### Estimating risk of autochthonous ZIKV transmission

To estimate the risk of autochthonous ZIKV transmission, we adopted the method described by Johansson et al. [34]. As ZIKV infection is a vector-borne disease, the basic reproduction number (R_0_), which is the average number of infected humans resulting from a single infected human in a completely susceptible population, is a product of two components - the average number of infectious vectors produced per infectious human (R_0_^HV^) and the average number of infectious humans produced per infectious vector (R_0_^VH^).

| $R_{0}= R_{0}^{\mathrm{HV}}R_{0}^{\mathrm{VH}}$ | (2.1) |
| --- | --- |

The parameter R_0_^HV^ can be derived by multiplying the number of female mosquitoes per person (*ψ*), the number of bites per mosquito per day (*α*), the effective transmission rate from human to vector (*β_HV_*), the average proportion of vectors surviving the extrinsic incubation period (*p_surv_*) and the mean duration of the human infectious period (*T_I_*).

| $R_{0}^{\mathrm{HV}}= \psi\alpha\beta_{HV} p_{surv} T_{I}$ | (2.2) |
| --- | --- |

The parameter R_0_^VH^ is the product of the effective transmission rate from vector to human (*β_VH_*), the average vector longevity (*L_v_*) and *α*.

| $R_{0}^{\mathrm{VH}}= \alpha\beta_{VH} L_{v}$ | (2.3) |
| --- | --- |

With R_0_^HV^ and R_0_^VH^, the probability of autochthonous transmission given the introduction of one seed (*p_autoone_*) can be calculated using the branching process approach as described by Johansson et al. [34]. In this approach, a single seeding case generates a random number of infectious vectors, which may go on to generate a random number of infectious humans. These generational steps can be characterized using negative binomial distribution-generating function, with dispersion parameter *k*. The probability-generating function for vectors can be described by *g_v_(s)*, where 0 ≤ *s* ≤ 1.

| $g_{V}(s)={[1+ \frac{R_{0}^{\mathrm{HV}}}{k} \left( 1-s \right)]}^{-k}$ | (2.4) |
| --- | --- |

Similarly, the probability-generating function for humans can be described by *g_H_(s)*.

| $g_{H}(s)={[1+ \frac{R_{0}^{\mathrm{VH}}}{k} \left( 1-s \right)]}^{-k}$ | (2.5) |
| --- | --- |

Combining Equation 2.4 and 2.5 results in a composite function for autochthonous transmission given the introduction of one seed *g_v_(g_H_(s)).*

| $g_{V}\left( g_{H}\left( s \right) \right)= {[1+ \frac{R_{0}^{\mathrm{HV}}}{k} \left( 1- g_{H}\left( s \right) \right)]}^{-k}$ | (2.6) |
| --- | --- |

As *g_v_(g_H_(s))* is a probability-generating function, the probability of extinction at zero generations can be estimated as *g(0)*. The parameter *p_autoone_* can hence be calculated as 1 - *g_v_(g_H_(0)).*

| $p_{autoone}=1-g_{V}\left( g_{H}\left( 0 \right) \right)$ | (2.7) |
| --- | --- |

Extending Equation 2.7, we can compute the probability of autochthonous transmission if there were independent introduction of multiple seeds.

| $Prob. of autochthonous transmission for introduction of multiple seeds ={1-g_{V}\left( g_{H}\left( 0 \right) \right)}^{No. of introduced seeds}$ | (2.8) |
| --- | --- |

- 1. **Varying proportion of population immune to ZIKV**

The probability of extinction after introduction of seeds increases if the human population is not fully susceptible due to prior exposure or cross-protection. To account for this, we replaced R_0_^VH^ with the effective number of infectious humans produced per infectious vector (R_E_^VH^) when estimating the effective reproduction number (R_E_) and probabilities of autochthonous transmission. The parameter R_E_^VH^ was derived by adjusting R_0_^VH^ with the proportion of human population who are immune (*prop_immune_*).

| $R_{E}^{\mathrm{VH}}= R_{0}^{\mathrm{VH}} {(1-prop}_{immune})$ | (2.9) |
| --- | --- |

The parameters used to compute the risk of autochthonous ZIKV transmission, and their definitions are summarized in Supplementary Table 1.

**Figures**

Supplementary Figure 1. Estimated mean number of ZIKV introductions from Latin America and the Caribbean into the United States (y-axis) versus ZIKV case counts reported by the United States Centers for Disease Control and Prevention (x-axis). Black vertical lines denote the 95% confidence intervals; black diagonal line denotes regression line; blue dotted lines denote the 95% confidence interval for the regression line; red diagonal line identifies the line y = x, where the estimated mean number of introductions equals the reported case counts*.*


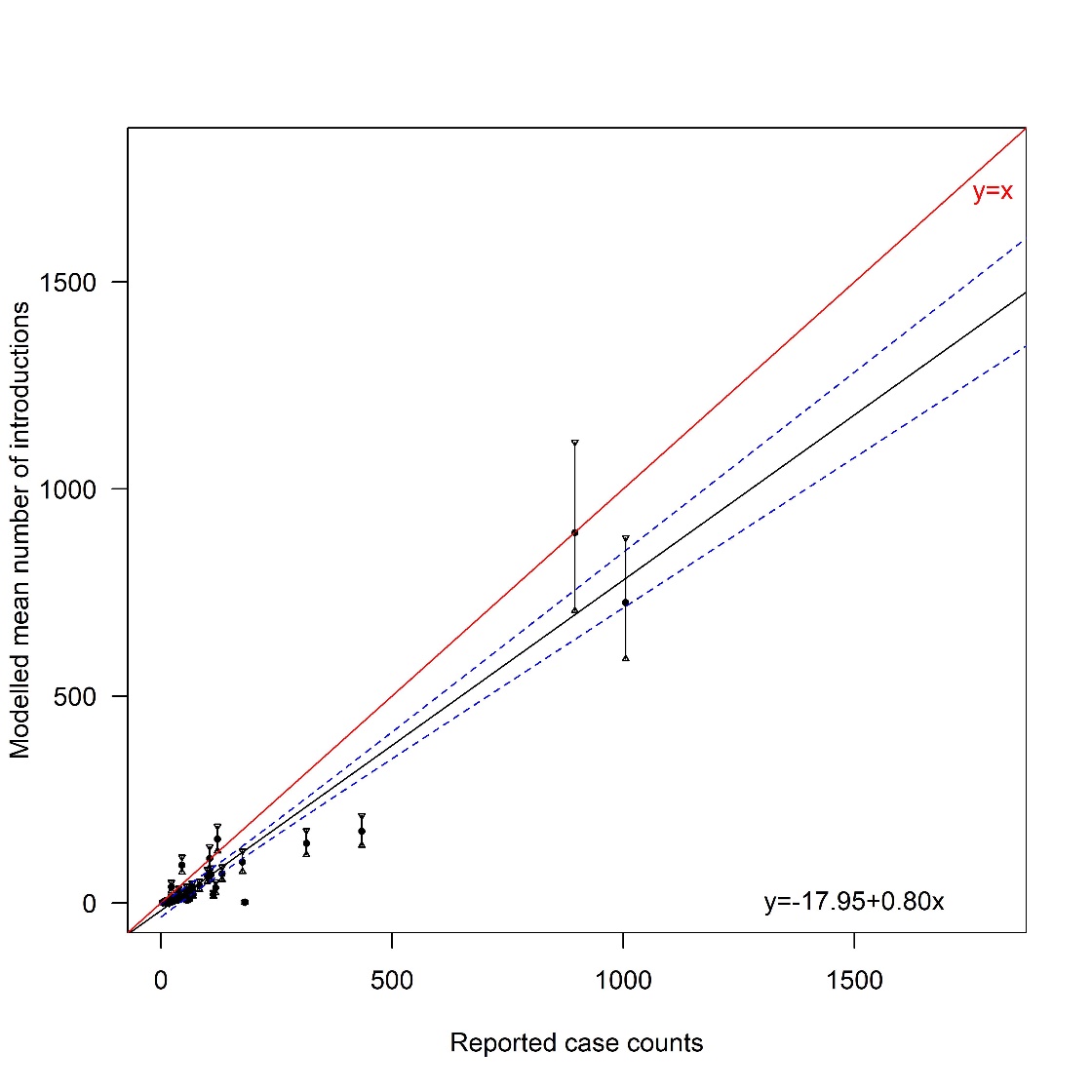


Supplementary Figure 2. Estimated probabilities of autochthonous transmission in Indonesia, Malaysia, the Philippines, Singapore, Thailand and Vietnam in the low transmissibility scenario assuming independent introductions of the estimated mean number of potential seeds at varying population immunity levels. Lines denote the estimated average probabilities of autochthonous transmission and the shaded areas denote the 95% confidence interval. Red, blue, black, green, gold colors respectively denote the dispersion parameter of k = 0.9, k = 0.5, k = 0.1, k = 0.05 and k=0.01.


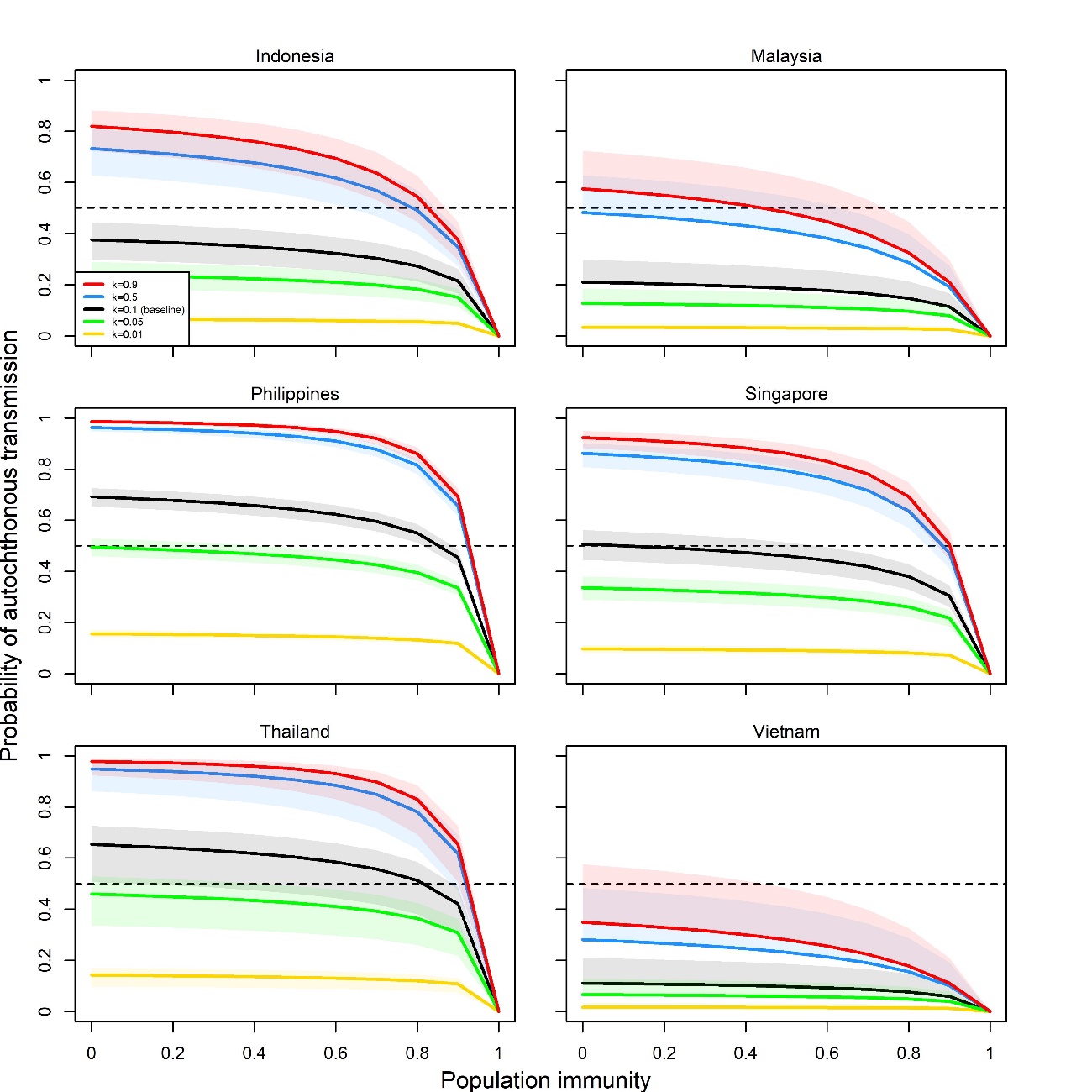


Supplementary Figure 3. Estimated probabilities of autochthonous transmission in Indonesia, Malaysia, the Philippines, Singapore, Thailand and Vietnam in the moderate transmissibility scenario assuming independent introductions of the estimated mean number of potential seeds at varying population immunity levels. Lines denote the estimated average probabilities of autochthonous transmission and the shaded areas denote the 95% confidence interval. Red, blue, black, green, gold colors respectively denote the dispersion parameter of k = 0.9, k = 0.5, k = 0.1, k = 0.05 and k=0.01.


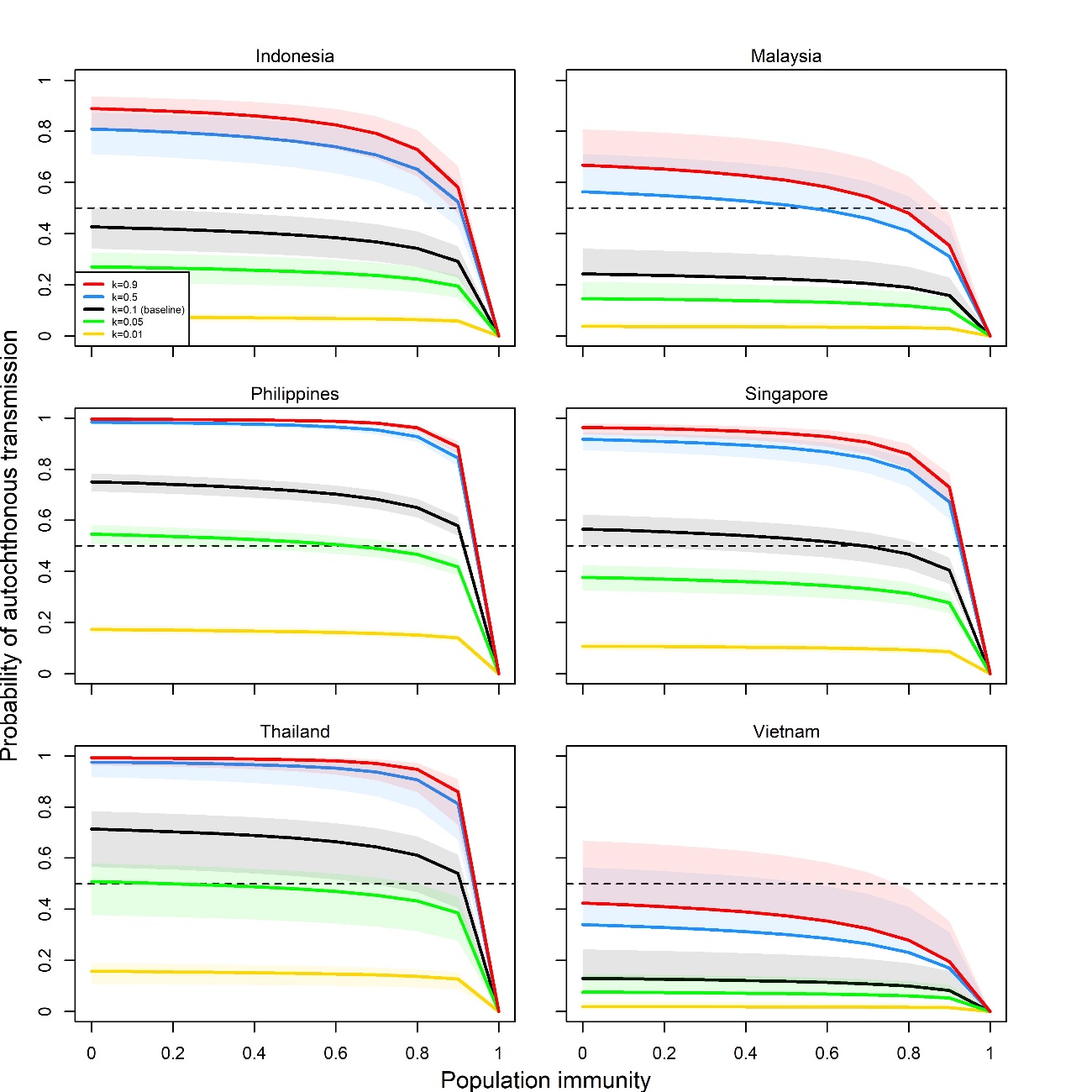


Supplementary Figure 4. Estimated probabilities of autochthonous transmission in Indonesia, Malaysia, the Philippines, Singapore, Thailand and Vietnam in the high transmissibility scenario assuming independent introductions of the estimated mean number of potential seeds at varying population immunity levels. Lines denote the estimated average probabilities of autochthonous transmission and the shaded areas denote the 95% confidence interval. Red, blue, black, green, gold colors respectively denote the dispersion parameter of k = 0.9, k = 0.5, k = 0.1, k = 0.05 and k=0.01.


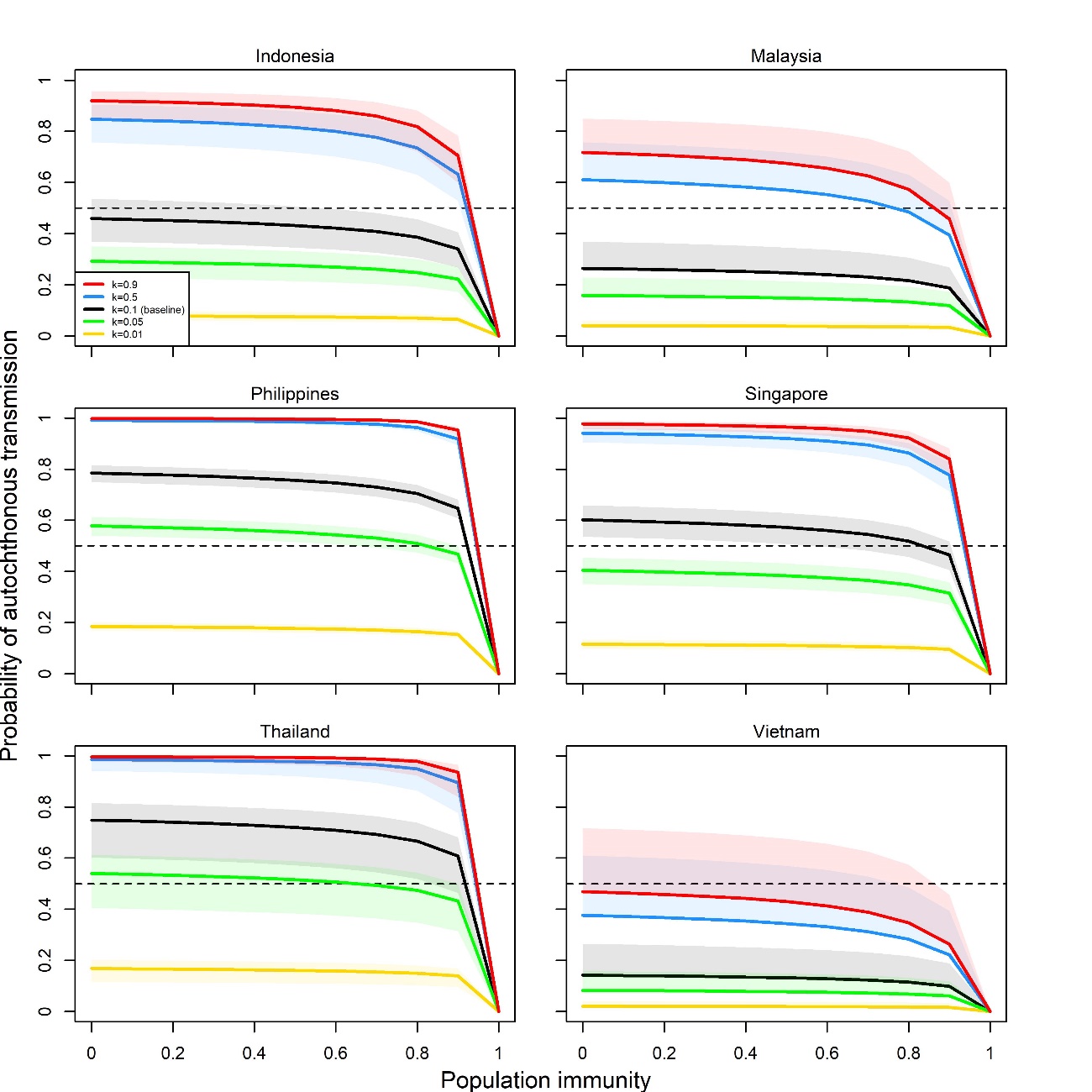


**Tables**

Supplementary Table 1. Parameterization used to estimate the risk of autochthonous Zika virus transmission and their definitions.

| Parameter | Definition |
| --- | --- |
| *ψ* | Number of female mosquitoes per person |
| *α* | Number of bites per mosquito per day |
| *T_I_* | Average duration of human infectious period (days) |
| *L_v_* | Average vector longevity (days) |
| *p_surv_* | Average proportion of vectors surviving the extrinsic incubation period |
| *β_HV_* | Effective transmission rate from human to vector |
| *β_VH_* | Effective transmission rate from vector to human |
| R_0_^HV^ | Average number of infectious vectors produced per infectious human in a completely susceptible population |
| R_0_^VH^ | Average number of infectious humans produced per infectious vector in a completely susceptible population |
| R_0_ | Basic reproduction number, average number of infected humans resulting from a single infected human in a completely susceptible population |
| R_E_ | Effective reproduction number, average number of infected humans resulting from a single infected human |

Supplementary Table 2. Estimated number of ZIKV introductions from Latin America and the Caribbean into each United States state and the reported case counts by the Centers for Disease Control and Prevention [22].

| States | Estimated number of introductions (mean and 95% confidence interval) | CDC-reported total number of Zika cases in 2015-16 | Number of introductions (after discarding cases acquired through local transmission) |
| --- | --- | --- | --- |
| Alabama | 7 (6–9) | 37 | 37 |
| Arizona | 20 (16-25) | 54 | 54 |
| Arkansas | 6 (5-7) | 15 | 15 |
| California | 173 (139-211) | 434 | 434 |
| Colorado | 33 (27-41) | 55 | 55 |
| Connecticut | 37 (25-52) | 118 | 118 |
| Delaware | 0 (0-0) | 17 | 17 |
| District of Columbia | 92 (74-111) | 45 | 45 |
| Florida | 895 (706-1,113) | 1,113 | 895 |
| Georgia | 69 (55-85) | 108 | 108 |
| Hawaii | 4 (3-4) | 15 | 15 |
| Idaho | 1 (1-1) | 5 | 5 |
| Illinois | 108 (84-136) | 105 | 105 |
| Indiana | 15 (12-39) | 50 | 50 |
| Iowa | 5 (4-6) | 26 | 26 |
| Kansas | 3 (2-3) | 20 | 20 |
| Kentucky | 8 (7-10) | 33 | 33 |
| Louisiana | 20 (16-24) | 38 | 38 |
| Maine | 5 (4-6) | 12 | 12 |
| Maryland | 71 (56-87) | 132 | 132 |
| Massachusetts | 154 (126-186) | 122 | 122 |
| Michigan | 39 (31-48) | 67 | 67 |
| Minnesota | 30 (24-38) | 68 | 68 |
| Mississippi | 3 (2-4) | 23 | 23 |
| Missouri | 29 (23-35) | 38 | 38 |
| Montana | 1 (1-2) | 9 | 9 |
| Nebraska | 6 (5-8) | 13 | 13 |
| Nevada | 39 (29-50) | 22 | 22 |
| New Hampshire | 2 (2-3) | 12 | 12 |
| New Jersey | 2 (1-2) | 181 | 181 |
| New Mexico | 4 (3-5) | 10 | 10 |
| New York | 726 (590-882) | 1,005 | 1,005 |
| North Carolina | 65 (52-81) | 100 | 100 |
| North Dakota | 1 (1-2) | 3 | 3 |
| Ohio | 42 (33-53) | 83 | 83 |
| Oklahoma | 9 (7-11) | 29 | 29 |
| Oregon | 10 (8-13) | 47 | 47 |
| Pennsylvania | 99 (76-125) | 176 | 176 |
| Rhode Island | 7 (5-9) | 56 | 56 |
| South Carolina | 13 (10-16) | 61 | 61 |
| South Dakota | 2 (1-2) | 3 | 3 |
| Tennessee | 23 (19-28) | 61 | 61 |
| Texas | 144 (117-175) | 320 | 314 |
| Utah | 13 (10-16) | 22 | 22 |
| Vermont | 3 (3-4) | 11 | 11 |
| Virginia | 21 (16-26) | 113 | 113 |
| Washington | 21 (16-26) | 69 | 69 |
| West Virginia | 1 (1-1) | 11 | 11 |
| Wisconsin | 12 (9-15) | 60 | 60 |
| Wyoming | 1 (1-1) | 3 | 3 |
